# BIOVERA-Tree: tree diversity, community composition, forest structure and functional traits along gradients of forest-use intensity and elevation in Veracruz, Mexico

**DOI:** 10.3897/BDJ.9.e69560

**Published:** 2021-09-09

**Authors:** María Leticia Monge González, Patrick Weigelt, Nathaly Guerrero-Ramírez, Dylan Craven, Gonzalo Castillo-Campos, Thorsten Krömer, Holger Kreft

**Affiliations:** 1 Biodiversity, Macroecology and Biogeography, University of Goettingen, Büsgenweg 1, 37077, Göttingen, Germany Biodiversity, Macroecology and Biogeography, University of Goettingen, Büsgenweg 1, 37077 Göttingen Germany; 2 Centro de Modelación y Monitoreo de Ecosistemas, Facultad de Ciencias, José Toribio Medina 29, Universidad Mayor, Santiago, Chile Centro de Modelación y Monitoreo de Ecosistemas, Facultad de Ciencias, José Toribio Medina 29, Universidad Mayor Santiago Chile; 3 Instituto de Ecología, A. C., Carretera antigua a Coatepec 351, 91070, Xalapa, Mexico Instituto de Ecología, A. C., Carretera antigua a Coatepec 351, 91070 Xalapa Mexico; 4 Centro de Investigaciones Tropicales, Universidad Veracruzana, José María Morelos 44 y 46, 91000, Xalapa, Mexico Centro de Investigaciones Tropicales, Universidad Veracruzana, José María Morelos 44 y 46, 91000 Xalapa Mexico

## Abstract

**Background:**

Here, we describe BIOVERA-Tree, a database on tree diversity, community composition, forest structure and functional traits collected in 120 forest plots, distributed along an extensive elevational gradient in Veracruz State, Mexico. BIOVERA-Tree includes information on forest structure from three levels of forest-use intensity, namely old-growth, degraded and secondary forest, replicated across eight elevations from sea-level to near the tree line at 3500 m and on size and location of 4549 tree individuals with a diameter at breast height ≥ 5 cm belonging to 216 species, 154 genera and 80 families. We also report measurements of eight functional traits, namely wood density for 143 species, maximum height for 216 species and leaf traits including: specific leaf area, lamina density, leaf thickness, chlorophyll content and leaf area for 148 species and leaf dry matter content for 145 species.

**New information:**

BIOVERA-Tree is a new database comprising data collected in a rigorous sampling design along forest-use intensity and elevational gradients, contributing to our understanding of how interactive effects of forest-use intensity and elevation affect tree diversity, community composition and functional traits in tropical forests.

## Introduction

Mountains are fascinating ecosystems and natural laboratories for evolutionary and ecological research as they encompass a wide variety of different climatic conditions over short distances (Körner 2004, Malhi et al. 2010). Mountains have captivated and inspired scientists since the seminal research by Alexander von Humboldt (von Humboldt 1806) and mountain research still contributes to our understanding of how environmental conditions affect plant distributions and how diversity may be impacted by global change (McCain and Grytnes 2010, Körner 2004, Malhi et al. 2010,Morueta-Holme et al. 2016). Mountains cover 25% of the Earth’s land surface and support an estimated one third of all terrestrial species (Körner 2004). Tropical mountains account for 10% of the terrestrial land area and are reservoirs of species diversity and hotspots of endemism with the potential to provide safe havens for species under current and predicted future anthropogenic global warming (Körner 2004, Sundqvist et al. 2013, Perrigo et al. 2020). Finally, tropical mountain forests provide a plethora of important ecosystem functions, for example, water storage and yield, carbon storage and pollination, that underpin ecosystem services and are a basis for human well-being (Díaz et al. 2018). Yet increasing human population and land-use intensification are altering forest structure, tree species diversity and functional diversity of tropical mountain forests around the world.

Land-use change and intensification are occurring at rapid rates and are strongly impacting mountain ecosystems (Payne et al. 2017). For instance, during a period of high deforestation in Mexico between 1980 and 2010, the State of Veracruz experienced the second highest rate of deforestation amongst all states, with 75% of its area being deforested (Ellis et al. 2011, Secretaría del Medio ambiente y recursos naturales 2016). Specifically between 1990 and 2000, Veracruz lost approximately 4.8% of its natural and secondary vegetation during this period, while only 8.6% of its vegetation remains conserved (Ellis et al. 2011). Afterwards, however, the region experienced a mild recovery in forest cover between 2000 and 2014 (Gómez-Díaz et al. 2018). Land-use change and intensification for timber extraction, agriculture and cattle pastures alter tree species diversity and community composition in this region (Monge‐González et al. 2020). Further, land-use change is not only a major threat to species diversity, but also has consequences for ecosystem functioning (Chapin et al. 2000, Newbold et al. 2015, Hooper et al. 2005). Therefore, high-quality databases of tree diversity and ecosystem functions are essential to understand the impacts of land-use change and elevation on tropical forests. Here, our objective is to contribute to the knowledge on how elevation and forest-use intensity interactively affect tropical tree diversity, community composition, functional traits and forest structure.

## General description

### Purpose

BIOVERA-Tree originated from the interdisciplinary research project BIOVERA, which aims at documenting and understanding biodiversity patterns along gradients of altitude, climate, soil and disturbance along an elevational transect at the Cofre de Perote in central Veracruz, Mexico (Carvajal‐Hernández et al. 2017, Gómez-Díaz et al. 2017, Bautista-Bello et al. 2019, Guzmán‐Jacob et al. 2020, Monge‐González et al. 2020). BIOVERA-Tree comprises forest plot data from eight elevational sites and three levels of forest-use intensity, namely old-growth, degraded and secondary forests. It contains descriptions of 120 non-permanent forests plots of 20 × 20 m and a community matrix including abundances for 216 species. Further, it contains measurements for 216 species including diameter at breast height (DBH) > 5 cm and tree height (metres) of 4548 and 4549 individuals, respectively. BIOVERA-Tree also includes functional traits for the common tree species, with data for wood density of 143 species calculated, based on 483 individuals, maximun height for 216 species calculated, based on 4549 individuals and leaf traits for 148 species, specific leaf area (n = 3148 leaves), lamina density (n = 3194 leaves), chlorophyll content (n = 3280 leaves), leaf area (n = 3214 leaves), leaf thickness (n = 3299) and leaf dry matter content for 145 species (n = 3081 leaves).

## Project description

### Study area description

**Figure 1.**
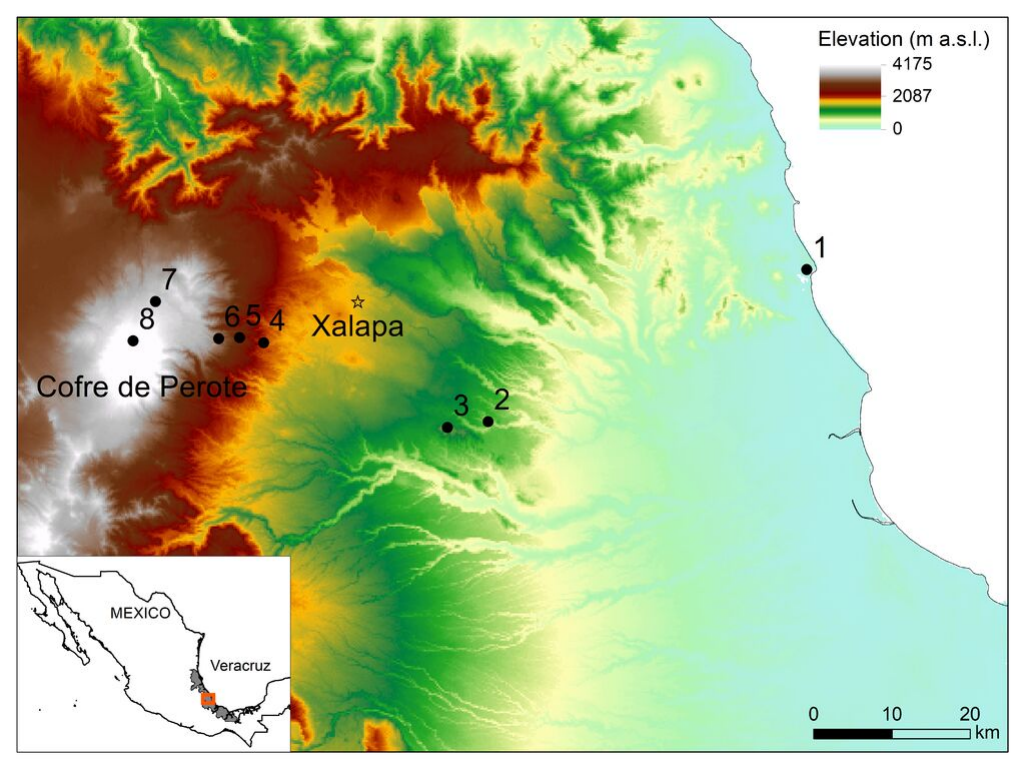
Location of the eight study sites along the elevational gradient at the eastern slopes of Cofre de Perote in Veracruz, Mexico. Black dots show the location of sites along the elevational gradient 1) 0 m; 2) 500 m; 3) 1000 m; 4) 1500 m; 5) 2000 m; 6) 2500 m; 7) 3000 m; 8) 3500 m.

The study area is located along an elevational gradient from sea level close to the Gulf of Mexico to near the tree line at 3545 m on the eastern slopes of the Cofre de Perote volcano, in the central part of the State of Veracruz, Mexico (Fig. 1). This region is located at the intersection of the Trans-Mexican volcanic belt and the Sierra Madre Oriental, resulting in complex geological conditions and is a transition zone where floristic elements from the Neotropics and Nearctic mix. Veracruz (including the study area) is part of the Mesoamerican biodiversity hotspot (Myers et al. 2000). The State harbours a diverse vascular flora of approximately 8500 species, which represents about 36% of the Mexican flora (Villaseñor 2016). The elevational gradient is characterised by a wide range of different environmental conditions. For instance, climate varies between tropical-dry at lower elevations, to temperate-humid at mid-elevations and cold-dry at high elevations (Soto-Esparza and Giddings-Berger 2011). The mean annual temperature ranges from 26°C near sea level to 9°C at the highest site. Mean annual precipitation varies between 1222 mm at low elevations, 2952 mm at mid-elevations and 708 mm at high elevations (Servicio Meteorológico Nacional 2019). Six main vegetation types along this elevation gradient are typically recognised, including tropical semi-humid deciduous, tropical oak, humid montane, pine-oak, pine and fir forests (Leopold 1950, Carvajal-Hernández et al. 2020). However, land-use change has altered these ecosystems into mostly degraded and secondary forests. The forests in the lowlands (0, 500 and 1000 m) have been largely replaced by agricultural systems, for example, sugar cane, corn, mango and lemon plantations and grasslands for cattle (Travieso-Bello et al. 2006, Thiébaut et al. 2017), while the forests at mid-elevations (1500, 2000 and 2500 m) have been transformed by illegal logging for charcoal production and firewood and converted into cattle pastures, coffee plantations and agricultural fields (Cruz-Angón et al. 2010) and the forests in the highlands (3000 and 3500 m) have been altered by timber extraction, agricultural fields for potatoes and broad beans, as well as pastures for goats and sheep (Pineda-López et al. 2013). Land-use modifications at every elevational site change with the primary economic activities.

## Sampling methods

### Sampling description

We selected eight sites along the elevational gradient, separated by about 500 m in elevation (Fig. 1). At each site, we established 15 plots of 20 × 20 m, with five plots located in old-growth, degraded and secondary forests, respectively (Suppl. material 1). This study design led to a total of 120 non-permanent forest plots (120 × 0.04 ha = 4.8 ha) inventoried along the elevation gradient.

Old-growth forests were defined as mature forests with low forest-use intensity and showed no signs of recent human use. Degraded forests were classified as intermediate forest-use intensity which had been subjected to selective logging and grazing by cattle or goats at high elevations. Finally, secondary forests were defined as high forest-use intensity, having regenerated following clear-cutting 15-20 years prior or with cattle grazing (Gómez-Díaz et al. 2017). In each plot, we inventoried all trees with a DBH > 5 cm and identified individual trees to the highest taxonomic resolution possible. For each individual, we measured its DBH (in cm) and tree height (in m), which was measured with a Leica laser (Homeier et al. 2010, Monge‐González et al. 2020). We calculated maximum tree height for each species following King et al. (2006) and classified the trees into three groups: 1) species with more than 20 individuals, 2) species with between 5-19 individuals and 3) species with less than five individuals. For the first group, we calculated maximum tree height as the mean of the tallest three individuals. For the second group, we estimated maximum tree height as the mean of the tallest two individuals; and for the third group, we used the height of the tallest individual (King et al. 2006).

In addition, we collected functional traits for the common tree species and we measured the following functional traits: maximum height, wood density, specific leaf area, leaf dry matter content, lamina density, leaf thickness, chlorophyll content and leaf area (Table 1). We selected these traits because they were expected to respond to both elevation and forest-use intensity (Díaz et al. 2016). For instance, the abundance of species with slow growth and conservative resource acquisition was expected to decrease, while that of species with fast growth and acquisition rates should increase with forest-use intensity (Table 1) (Lavorel and Garnier 2002, Díaz et al. 2016). For trait measurements, we selected one to three tree individuals per species and collected at least five to ten leaves per individual and one to three wood cores per species along the gradients of elevation and forest-use intensity.

For wood density, we collected wood samples using an increment borer. We used the water-displacement method for measuring wood sample volume and oven-dried samples at 70°C for 48 to 72 hours until they reached a constant dry weight (Chave 2005). We determined maximum height for each species following King et al. (2006). For leaf traits, we followed standardised trait measurement protocols (Pérez-Harguindeguy et al. 2016). We weighed the fresh leaves and then oven-dried them at 60°C for 48 hours or until they had reached a constant dry weight. We measured leaf thickness with a digital caliper. For chlorophyll concentration, we used a SPAD-502 chlorophyll meter (Spectrum Technologies, Plainfield, IL, USA) and converted measurements to chlorophyll concentration following (Coste et al. 2010). We calculated leaf area using WinFOLIA (Version 2016b Pro, Regent Instruments Canada, 2016). In total, we collected wood samples for 143 species and leaf samples for 148 species, except leaf dry matter content, for which we have samples for 145 species. We followed the definition and categories according to Darwin Core, Functional Diversity thesaurus (Suppl. material 6).

## Geographic coverage

### Description

The BIOVERA elevational gradient is located close to the Gulf of Mexico and spans from close to sea level (19.5894N, -96.375167W) to close to the treeline at 3545 m elevation (19.5182N, -97.154525W) along the eastern slopes of Cofre de Perote volcano (4282 m) in Veracruz State, Mexico (Fig. 1).

## Taxonomic coverage

### Description

Taxonomic information on valid species, genus and family names was obtained from The Plant List version 1.1 (2013). Individuals were identified to the species level by specialists (Dr. Francisco Lorea-Hernández, M.Sc. Claudia Gallardo-Hernández and Biol. Carlos M. Durán-Espinosa, Instituto de Ecología, A. C.), while some individuals could only be identified to the family or genus level or could not be identified. Vouchers of specimens were deposited at the Herbarium XAL of Instituto de Ecología, A.C. at Xalapa, Mexico.

**Tree diversity and community composition**

The database contains information of 216 tree species (Suppl. material 2) distributed amongst 80 families and 154 genera and tree abundances across plots along the forest-use intensity and elevation gradients (Suppl. material 3, Suppl. material 4). We recorded a total of 4,549 individual trees, we identified 4136 individuals to species, 307 individuals were identified to genus, 57 individuals were identified to family and 49 individual trees were not identified. The number of species per plot ranged from 1 to 18, with a mean 8.1 species (Table 2).

**Figure 2.**
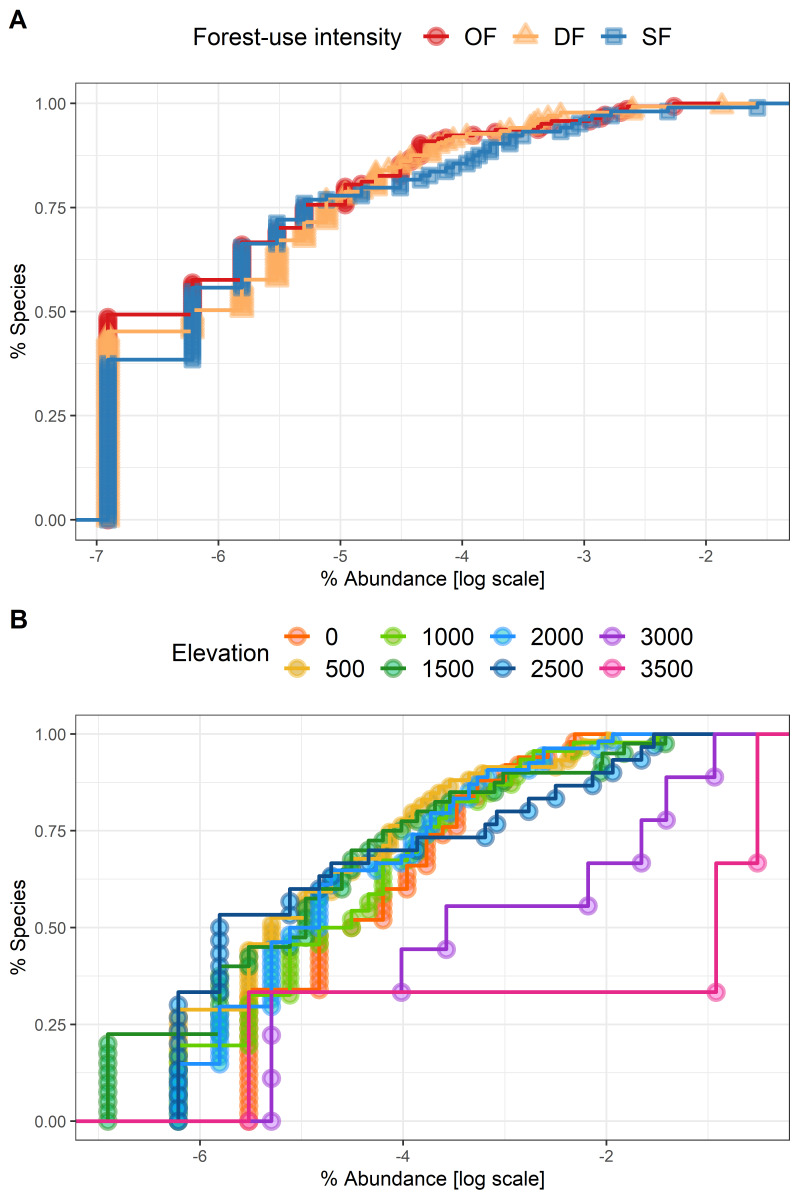
Species-abundance distributions (n = 120 plots) using an empirical cumulative distribution function **A** for different levels of forest-use intensity and **B** for eight sites along the elevation gradient. Vertical axis shows each species from most to least abundant. Horizontal axis shows the relative abundance of the species on a logarithmic scale. Forest-use intensity levels are old-growth forest (OF), degraded forest (DF) and secondary forest (SF).

The number of individuals per plot ranged from 4 to 120 with a mean of 8.19 individuals (Table 2). Species-abundance distributions across levels of forest-use intensity indicated a higher proportion of rare species in old-growth and degraded forests than in secondary forests (Fig. 2A). Species-abundance distributions across elevations revealed that forests at high elevations (3000-3500 m) are dominated by a small number of species, while forests at low elevations (0-1000 m) and mid-elevations (1500-2000 m) exhibited higher evenness (Fig. 2B).

**Forest structure**

**Figure 3.**
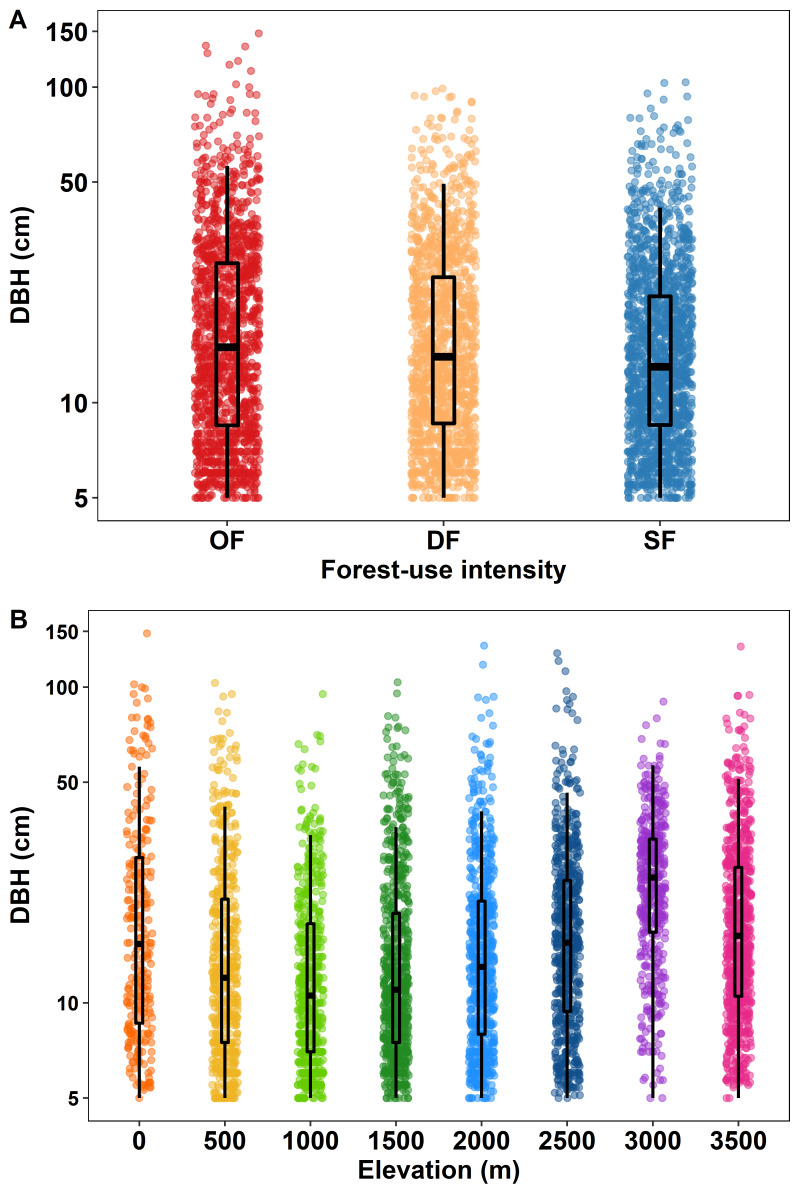
Diameter at breast height (DBH; n = 4127 individuals) for **A** different levels of forest-use intensity and for **B** eight sites along the elevation gradient. Vertical axis shows tree diameter at breast height (DBH) on a logarithmic scale. Forest-use intensity levels are old-growth forest (OF), degraded forest (DF) and secondary forest (SF). Boxes are second and third quartile, whiskers upper and lower quartile and horizontal lines indicate mean values.

Across all plots, DBH ranged from 5 to 148 cm, with a mean of 14 ± 15.5 SD (Fig. 3). DBH mean per forest-use intensity varied from 20.84 ± 17.6 SD in old-growth forests, 19.3 ± 15.2 SD in degraded forests and 17.4 ± 13.3 SD in secondary forests. The four species with the highest mean DBH were *Pseudobombaxellipticum*, *Salixhumboldtiana*, *Diphysarobinioides* and *Pachiraaquatica*.

## Traits coverage

**Tree functional traits**

**Figure 4.**
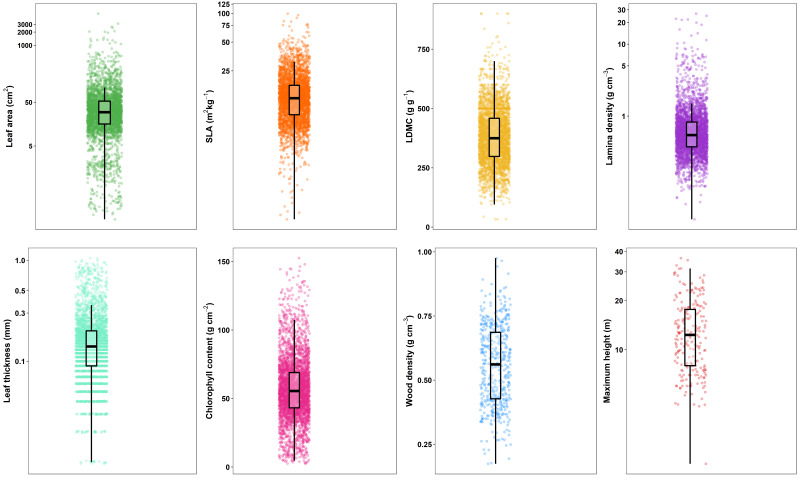
Distribution of eight functional traits along elevation and forest-use intensity gradients in the BIOVERA-Tree. Points represent leaf-level data for specific leaf area (n = 3148), leaf dry matter content (n = 3081), lamina density (n= 3194), leaf area (n = 3214), leaf thickness (n = 3299), and chlorophyll content (n = 3280); individual-level data for wood density (n = 483); and species-level data for maximum height (n = 216). Boxes are second and third quartile, whiskers upper and lower quartile and horizontal lines indicate mean values.

This dataset contains eight functional traits (Table 1, Fig. 4). The number of species per functional trait varies, from 216 species for maximum height, to 143 for wood density and 143- 148 species for leaf traits.

## Usage licence

### Usage licence

Creative Commons Public Domain Waiver (CC-Zero)

## Data resources

### Data package title

Data package title BIOVERA-Tree: community, functional traits and forest structure along forest-use intensity and elevational gradients in Veracruz, Mexico.

### Number of data sets

6

### Data set 1.

#### Data set name

BIOVERA-Tree forest plots description

#### Number of columns

13

#### Description

Location of the 120 plots along the elevational gradient at the eastern slopes of Cofre de Perote in Veracruz, Mexico. Available as Suppl. material 1.

**Data set 1.** 

Column label	Column description
locationID	An identifier for the set of location information (data associated with dcterms:Location). May be a global unique identifier or an identifier specific to the dataset.
roundElevation	round metres above sea level
verbatimElevation	The original description of the elevation (altitude, usually above sea level) of the Location.
habitat	A category or description of the habitat in which the Event occurred.
forestUseIntensity	Old growth forest (OF) a mature forest with low forest-use intensity, degraded forest (DF) classified as intermediate forest-use intensity and secondary forest (SF) high forest-use intensity
verbatimLatitude	The verbatim original latitude of the Location. The coordinate ellipsoid, geodeticDatum or full Spatial Reference System (SRS) for these coordinates should be stored in verbatimSRS and the coordinate system should be stored in verbatimCoordinateSystem.
verbatimLongitude	The verbatim original longitude of the Location. The coordinate ellipsoid, geodeticDatum or full Spatial Reference System (SRS) for these coordinates should be stored in verbatimSRS and the coordinate system should be stored in verbatimCoordinateSystem.
temperature	Mean annual temperature (in degrees Celsius)
precipitation	Mean annual precipitation (in millimetres)
country	The name of the country or major administrative unit in which the Location occurs.
eventDate	The date or interval during which an Event occurred. For occurrences, this is the date when the event was recorded.
coordinateUncertaintyInMetres	The horizontal distance (in metres) from the given decimalLatitude and decimalLongitude describing the smallest circle containing the whole of the Location.
geodeticDatum	The ellipsoid, geodetic datum or spatial reference system (SRS) upon which the geographic coordinates given in decimalLatitude and decimalLongitude are based. Here: WGS84

### Data set 2.

#### Data set name

BIOVERA-Tree scientific name

#### Number of columns

3

#### Description

List of tree species along the elevational gradient and different levels of forest-use intensity. Available as Suppl. material 2.

**Data set 2.** 

Column label	Column description
treeIdentificationID	An identifier for the nomenclatural (not taxonomic) details of a scientific name.
treeIdentification	The full scientific name, with authorship and date information, if known. When forming part of an Identification, this should be the name in lowest level taxonomic rank that can be determined.
family	The full scientific name of the family in which the taxon is classified

### Data set 3.

#### Data set name

BIOVERA-Tree community matrix

#### Number of columns

2

#### Description

Tree community matrix composition along eight elevational sites and three different forest-use intensity levels of 216 tree species (n = 5 plots per forest-use intensity within elevation). The numbers within the matrix are the number of individuals. Available as Suppl. material 3.

**Data set 3.** 

Column label	Column description
locationID	Forest plot identifier from supplementary table 1 (rows)
treeIdentificationID	Scientific name identifier from supplementary table 1 (columns)

### Data set 4.

#### Data set name

BIOVERA-Tree forest structure

#### Number of columns

5

#### Description

Diameter at breast height (DBH) > 5 cm and tree height (metres) for 216 species along the elevational gradient and different levels of forest-use intensity. Available as Suppl. material 4.

**Data set 4.** 

Column label	Column description
locationID	An identifier for the set of location information (data associated with dcterms:Location). May be a global unique identifier or an identifier specific to the dataset.
treeIdentificationID	An identifier for the nomenclatural (not taxonomic) details of a scientific name.
organismID	An identifier for the Organism instance (as opposed to a particular digital record of the Organism). May be a globally unique identifier or an identifier specific to the dataset.
variableName	name of the variable
variableValue	value

### Data set 5.

#### Data set name

BIOVERA-Tree functional traits

#### Number of columns

7

#### Description

Plant functional traits measured along the elevational gradient and different levels of forest-use intensity; including leaf traits, wood density and maximum height. Available as Suppl. material 5 .

**Data set 5.** 

Column label	Column description
treeIdentificationID	An identifier for the nomenclatural (not taxonomic) details of a scientific name.
organismID	An identifier for the Organism instance (as opposed to a particular digital record of the Organism). May be a globally unique identifier or an identifier specific to the dataset.
leafID	An identifier for leaf
heightGroup	It is the classification of trees into three groups: 1) species with more than 20 individuals, 2) species with between 5-19 individuals and 3) species with less than five individuals.
resolution	Scale
traitName	Functional trait
traitValue	Functional trait value

### Data set 6.

#### Data set name

BIOVERA Tree metadata

#### Number of columns

3

#### Character set

metadata

#### Description

Definition and categories according with Darwin Core, Functional Diversity thesaurus and this research.

**Data set 6.** 

Column label	Column description
According	Type of terminological resource for plant characteristics.
Concept	Term, name of the variable
Definition	Explanation of concepts and variables

## Additional information

The data underpinning the analysis reported in this paper are deposited in the Dryad Data Repository at https://doi.org/10.5061/dryad.ngf1vhhvb.

## Supplementary Material

1BCEFC74-C760-5C41-BC74-77C909F237EB 10.3897/BDJ.9.e69560.suppl1 Supplementary material 1 BIOVERA-Tree forest plots description Data type Location Data File: oo_549009.csv https://binary.pensoft.net/file/549009 María Leticia Monge-González, Patrick Weigelt, Nathaly Guerrero-Ramírez, Dylan Craven, Gonzalo Castillo-Campos, Thorsten Krömer, Holger Kreft.

297517E1-0C9F-5503-B9D1-6B484B74C9E7 10.3897/BDJ.9.e69560.suppl2 Supplementary material 2 BIOVERA-Tree scientific name Data type Taxonomy File: oo_549044.csv https://binary.pensoft.net/file/549044 María Leticia Monge-González, Patrick Weigelt, Nathaly Guerrero-Ramírez, Dylan Craven, Gonzalo Castillo-Campos, Thorsten Krömer, Holger Kreft.

42D688AD-8284-53B7-8D58-28F37D03F2EF 10.3897/BDJ.9.e69560.suppl3 Supplementary material 3 BIOVERA-Tree community matrix Data type Species community data Brief description Tree community matrix with abundances File: oo_549021.csv https://binary.pensoft.net/file/549021 María Leticia Monge-González, Patrick Weigelt, Nathaly Guerrero-Ramírez, Dylan Craven, Gonzalo Castillo-Campos, Thorsten Krömer, Holger Kreft.

1B6E2702-97B7-5634-BDC4-A09F03A37092 10.3897/BDJ.9.e69560.suppl4 Supplementary material 4 BIOVERA-Tree forest structure Data type Forest structure, DBH, tree height File: oo_549014.csv https://binary.pensoft.net/file/549014 María Leticia Monge-González, Patrick Weigelt, Nathaly Guerrero-Ramírez, Dylan Craven, Gonzalo Castillo-Campos, Thorsten Krömer, Holger Kreft.

8880BA0C-CC51-5419-83D8-EA722FAC0545 10.3897/BDJ.9.e69560.suppl5 Supplementary material 5 BIOVERA-Tree functional traits Data type Functional trait data File: oo_549015.csv https://binary.pensoft.net/file/549015 María Leticia Monge-González, Patrick Weigelt, Nathaly Guerrero-Ramírez, Dylan Craven, Gonzalo Castillo-Campos, Thorsten Krömer, Holger Kreft.

BB3E33BB-DDFE-53F9-976F-4676697DF15D 10.3897/BDJ.9.e69560.suppl6 Supplementary material 6 BIOVERA-Tree metadata Data type metadata Brief description Definition and categories according with Darwin Core, Functional Diversity thesaurus and this research. File: oo_549022.csv https://binary.pensoft.net/file/549022 María Leticia Monge-González, Patrick Weigelt, Nathaly Guerrero-Ramírez, Dylan Craven, Gonzalo Castillo-Campos, Thorsten Krömer, Holger Kreft.

## Figures and Tables

**Table 1.** Number of individuals and species with measurements of eight functional traits.

**Ecological relevance**	**Functional Trait**	**Unit**	**Measured individuals**	**Number of species**
Seed dispersion, competitive ability	Maximum height	m	4549^†^	216
Structure and mechanical support	Wood density	g cm-^3^	483	143
Leaf energy and water balance, physical strength	Specific leaf area	m^2^ kg^-1^	3148	148
	Leaf dry matter content	g g^-1^	3081	145
	Leaf thickness	mm	3299	148
	Lamina density	g cm^-3^	3194	148
Photosynthesis	Leaf area	cm^2^	3214	148
	Chlorophyll content	μg cm^−2^	3280	148

**Table 2.** Mean tree species and individual numbers per plot.

**Elevation (m)**	**Species(mean** ± **SD)**	**Individuals(mean** ± **SD)**
0	6.8 ± 4.16	17.46 ± 8.68
500	11.06 ± 3.63	36.4 ± 11.91
1000	13.33 ± 2.22	35.4 ± 12.7
1500	9.6 ± 4.56	44.73 ± 15.9
2000	13.13 ± 2.94	44.13 ± 10.4
2500	6.8 ± 2.99	42.20 ± 13.2
3000	3.06 ± 0.96	28.93 ± 10.5
3500	1.66 ± 0.72	51.4 ± 30.81
